# A comparative observational study of carbohydrate intake and continuous blood glucose levels in relation to performance in ultramarathon

**DOI:** 10.1038/s41598-023-51048-6

**Published:** 2024-01-11

**Authors:** Naho Inamura, Hirokazu Taniguchi, Shiori Yoshida, Masato Nishioka, Kengo Ishihara

**Affiliations:** 1 Faculty of Agriculture, Ryukoku University, Shiga, 520-2194 Japan; 2 Division of Applied Life Sciences, Graduate School of Life and Environmental Sciences, Kyoto Prefectural University, Kyoto, Japan

**Keywords:** Nutrition, Weight management, Nutritional supplements, Metabolism

## Abstract

Ultra-endurance events have gained global participation, whereas the critical factors of competition results remain to be well elucidated. This study used a nutritional approach to evaluate the association of competition results with carbohydrate intake and blood glucose control during a 100-mile ultramarathon. This observational study was conducted in the 2021 LAKE BIWA 100, which covered 100 miles (169 km) and 10,500 m elevation. The course was divided into 9 segments by aid station. According to the competition results, 22 participants (18 men and 4 women) were divided into higher finishers (*n* = 7), lower finishers (*n* = 9), and non-finishers (*n* = 6). The participants self-recorded their overall dietary intake throughout the race. Glucose levels were monitored every 15 min by a flash glucose monitoring system. Running speed in each segment was standardized to the average of the top five finishers for each gender. Among finishers, the carbohydrate intakes were significantly higher in the higher finishers than in the lower finishers during overall segments, especially in the first half of the race (*p* < 0.05). There was a significant positive correlation between running speed and carbohydrate intake in the lower finishers (*rho* = 0.700, *p* = 0.036). Two-way ANOVA analysis revealed that lowering glucose levels in each segment were more frequently observed in the lower finishers compared to the higher finishers (*p* = 0.012). Compared to the higher finishers, the lower finishers exhibited significantly greater fluctuations (⊿highest-lowest) in glucose levels (*p* < 0.001). The fluctuations in glucose levels were significantly and negatively correlated with the running speed of the finishers (*rho* =  − *0.612, p* = *0.012*). Faster runners consume high amounts of carbohydrates and maintain glucose levels during the 100-mile ultramarathon on the trail, especially at the beginning. Lowering and fluctuating glucose levels during the race are associated with lower running speed in endurance athletes.

## Introduction

Ultra-endurance events, which are greater than 6 h in duration^1^ have gained substantial popularity and global participation^2,3^. Ultramarathon runs on trails are the most popular ultra-endurance events^4^, and the recent increase in these events implies an increase in both competitive and recreational participants^2,3^. Although it is thought that ultramarathon performance was determined by physiological parameters such as maximal oxygen uptake and the energy cost of running ^4,5^, the key physiological variables has not been confirmed for longer-distance races more than 100 km^6–8^. Therefore, comprehending the method to enhance performance and complete the full distance in the ultramarathon events is important for these participants, whereas the determinant factors have not been fully understood.

Energy expenditure during ultramarathon was estimated at more than 10,000 kcal per day^9,10^, suggesting that the energy compensation by macronutrient intake is regarded as a determinant factor of ultra-endurance performance^10,11^. Indeed, the total energy intake during ultramarathon was reported to be associated with faster running speed^12^, and was higher in the finishers than in the non-finishers^13,14^. Because the primary energy substrate during exercise is carbohydrate^15,16^, endurance athlete has been recommended to consume 90 g/hour of carbohydrate during exercise^17^. Our previous research indicated that amount of carbohydrate intake during ultramarathon was positively correlated with faster running speed in 7 finishers^12^.

The reason why carbohydrate intake improves long-term endurance performance is classically considered as maintaining muscle glycogen and blood glucose levels^18,19^. The agreement between the classical finding and ultramarathon performance was confirmed using a continuous glucose monitoring system that indicated that a decrease in the glucose level was associated with lower running speed during an ultramarathon in several finishers^12,20^. The monitoring system also revealed that the increased fluctuations in glucose levels decreased running speed^12^. Therefore, it is considered that maintaining glucose levels during the race is associated with ultra-endurance performance.

However, to the best of our knowledge, it has not been well evaluated whether carbohydrate intake and glucose control during ultramarathon are different among competition levels, including endurance performance and completing the race. We hypothesized that participants who had lower level of competition showed inadequate carbohydrate supplementation, lower monitoring glucose levels, and higher glucose fluctuations during the race. Thus, this study collected data on dietary intake using the self-recording method and glucose levels using a flash glucose monitoring system (FGM), thereby evaluating the association of competition results with carbohydrate intake and glucose control during a 100-mile ultramarathon.

## Methods

### Study design

This observational study was designed to investigate the relationship between running speed, dietary intake, and monitored glucose levels during an ultramarathon. All procedures were approved by the Ryukoku University Human Research Ethics Review Board (No. 2021-21). Written informed consent was obtained from all the participants before enrollment in the study. The study was conducted in accordance with the Declaration of Helsinki.

### Race course and runners

The present study was conducted in the 2021 LAKE BIWA 100^21^, performed on October 1–3, 2021, in Shiga, Japan. The distance of the course covered 100 miles (169 km), and the total elevation was 10,500 m. The course included trails, rocks, paths, grasslands, and pavements. The course was divided into 9 segments by eight aid stations where each runner's passing time was recorded electronically. All runners had global positioning system (IBUKI GPS; OND Inc., Japan) throughout the ultramarathon to record location data and running speed. Distances between each aid station were 18.8 ± 7.3 km and varied from 7 to 28 km. The time limit was 52 h.

All the runners had completed the ultramarathon race, and the sum of points certified by the International Trail Running Association exceeded 6 in the last three years, demonstrating their experience in ultra-endurance events. Thus, only total 100 ultra-endurance athlete (86 men and 14 women) who had a high endurance capacity could participate the 2021 LAKE BIWA 100. All the runners had to run with backpacks to carry necessities, including dietary intake and drink. Finally, 77 runners (77%) completed the 100-mile ultramarathon, and the median finish time was 45:15 (hours:minutes). On the other hand, 23 runners could not finish the event.

### Study participants and groups

Twenty-two participants (18 men and 4 women) out of the overall 100 runners voluntarily participated in the present study. Participant recruitment was through advertisement by organizer of the event and by personal social media. Sex, height, and weight were self-reported, and body mass index (BMI) was calculated by the standard formula. In the ultramarathon study, 16 participants (72.7%) completed the full distance, and the remaining participants did not finish (DNF, *n* = 6). The retirements were observed after segments of 3 (47 km, *n* = 1), 4 (75 km, *n* = 2), 5 (97 km, *n* = 2), and 7 (125 km, *n* = 1). The DNF was caused by completion time limit (52 h), and severe injury was not confirmed in the participants. The ranges of DNF time were from 18:37 to 36:08. According to the median overall finish time, the 16 finishers were divided into the higher group (*n* = 7) and lower group (*n* = 9). The ranges of finish time were from 28:08 to 43:31 in the higher group and from 47:11 to 50:41 in the lower group.

### Running speed and standardization

Running time and speed between each aid station were obtained from the official website^21^. As shown in previous studies^12^, the standard running speed for each segment was calculated by averaging the top five finishers according to sex. Running speed was expressed as running distance/hour (km/h), and the standardized running speed for each segment was calculated for each male and female participant. The running speed of runners in each segment was standardized using the following formula: %Running speed = (The participant's running speed) / (Average of top 5 finishers' running speed in each sex) × 100. The standardized running speed exceeds 100% only when running at a pace comparable to the top 1 and 2 places in each sex.

### Dietary data

Participants self-recorded their overall timing and volume of food intake and drink consumption throughout the ultramarathon. Food and drink consumed more than 60 min before the start were not included in the dietary data. Total food consumption was confirmed by pictures taken before and after the race. The dietary intakes during the race were calculated from the nutrition information of products. If the data were unavailable, these nutritional intakes were calculated by the standard tables of food composition in Japan 2020^22^. They were expressed as per body weight (kg)/running time (hours) ^12,14^.

Habitual dietary data were obtained using brief-type self-administered diet history questionnaire (BDHQ)^23,24^. The BDHQ is a self-administered questionnaire, which assess food consumption frequency in the past month. Energy and macronutrient intakes were calculated by dietary intakes for food and beverage items^23,24^.

### Glucose data and standardization

Circulating glucose levels were monitored by FGM, as described by other studies^25,26^. In Brief, the FGM system (FreeStyle Libre Pro; Abbott Diabetes Care, Alameda, CA) continuously measures glucose concentration in the interstitial fluid below the skin, and produces the corresponding ambulatory device. The FGM sensor was applied at the back of the upper arm, and glucose concentrations were obtained every 15 min. Participants were attached to the device more than 24 h before the start of ultramarathon start.

**Figure 1:**
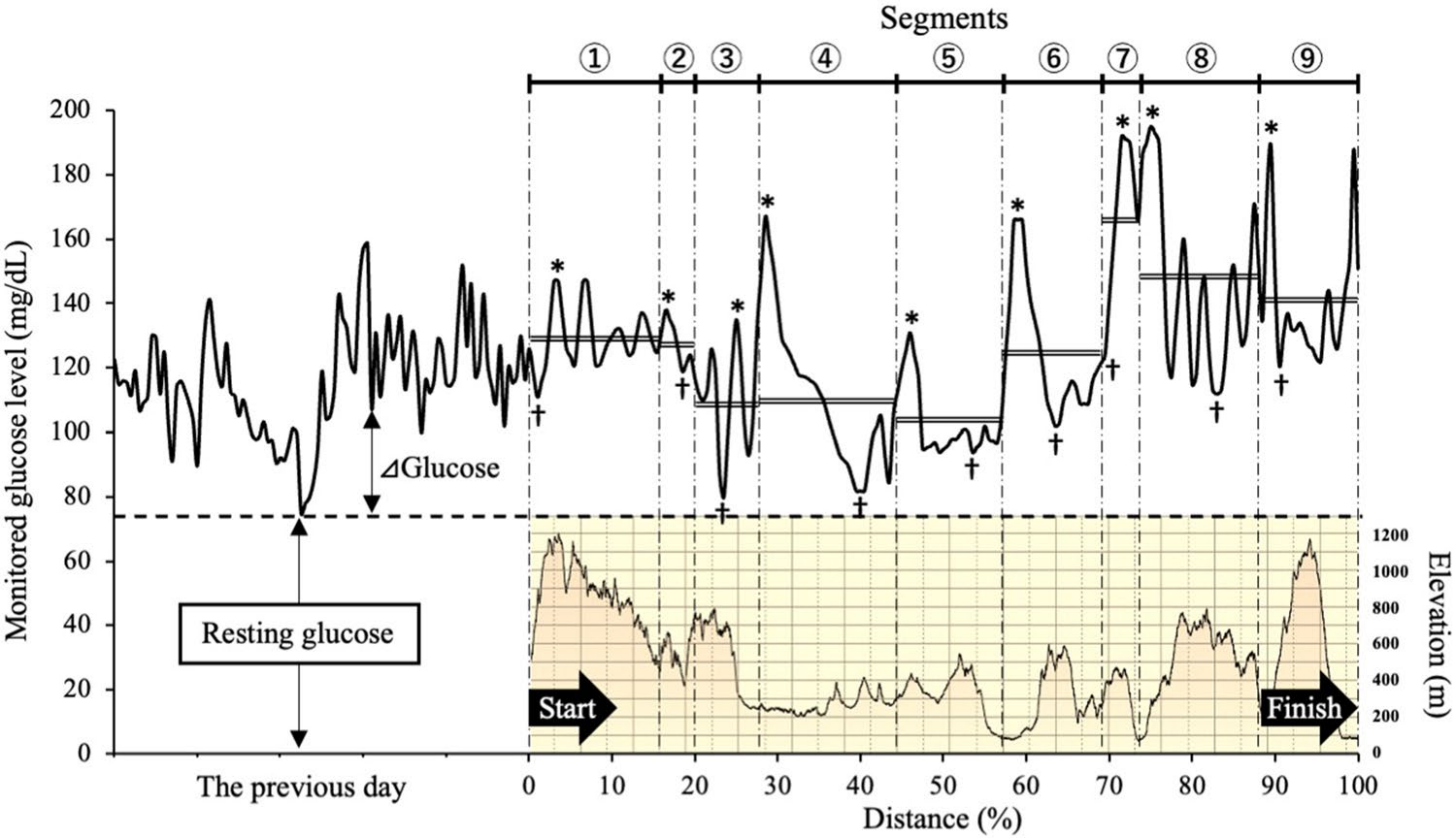
Standardization of glucose levels after and during a 100-mile ultramarathon. The course was divided into 9 segments (① ~ ⑨, vertical dashed line) by 8 aid stations. The altitude profile is shown on the right vertical axis and filled area. A representative result of monitoring glucose levels is shown on the left vertical axis and solid line. ⊿Glucose level was expressed by subtracting the resting glucose level in each runner (horizontal dashed line). In each segment, * indicates the highest ⊿glucose, † indicates the lowest ⊿glucose, and double lines indicate average ⊿glucose levels.

For each participant, the glucose concentration during the race was standardized by subtracting the resting fasting glucose concentration (Fig. 1). Thus, the glucose levels were expressed as an increase from the resting fasting glucose level (⊿glucose). In addition to the average, highest, and lowest ⊿glucose levels, the difference between the highest and lowest glucose levels in each participant was calculated in each segment.

### Statistics

All statistical analyses were performed using SPSS version 29.0 (SPSS, Inc., Chicago, IL). The statistical power of one-way ANOVA was calculated based on 3 group with 6 participants each (total 18 participants), difference of 0.1 g/kg/h carbohydrate intake among the groups, 0.5 standard deviation, and 0.05 significance level. The calculated statistical power was 0.81. The Kolmogorov–Smirnov test was performed to assess the normality of data distribution. Differences in continuous variables among the higher group, lower group, and DNF group were assessed by one-way ANOVA followed by Tukey's HSD post-hoc test. Kruskal–Wallis test with Bonferroni correction was used for non-parametric data. Categorical data were analyzed using the chi-square test. Changes in carbohydrate intake and monitoring glucose levels during the ultramarathon were analyzed by two-way ANOVA with Tukey's post hoc test. The two-way ANOVA (group x segment) was performed throughout the race excluding the DNF group, and from segment 1–3 including all groups. For dietary intakes, a difference between the first half and last half of the race was examined using the two-way ANOVA test. Associations of dietary intake and glucose control with running speed were determined by Spearman's rank correlation coefficient. The data are presented as means and standard deviations. The level of statistical significance was set at *p* < 0.05.

## Results

### Participant characteristics

There were no statistical differences in sex, height, weight, BMI, and resting glucose level among the groups (Table 1). However, the DNF group was significantly older in age than the higher and lower groups (*p* < 0.05). The higher group consumed significantly higher amounts of fat and carbohydrate g/kg/h during the race compared to the lower group. Carbohydrate intake per time was not significantly different among the groups. For standardized glucose levels (⊿glucose), the difference between the highest and lowest glucose in each segment was significantly lower in the higher group than in the lower group (*p* < 0.05).

**Table 1:** Comparison of participant characteristics, glucose level, dietary intake, and running speed among the groups.

	Overall (*n* = 22)				Higher (*n* = 7)			Lower (*n* = 9)			DNF (*n* = 6)			*p*
Sex (men/women)	18/4				6/1			7/2			5/1			0.914
Age (years)	46.0	±	6.9		40.6	±	6.9^b^	45.8	±	4.6^b^	52.8	±	3.4^a^	**0.002**
Height (cm)	169.1	±	7.6		173.7	±	6.8	168.5	±	5.5	164.7	±	9.4	0.097
Weight (kg)	60.3	±	6.6		62.8	±	5.9	60.3	±	6.0	57.2	±	7.9	0.328
Body mass index(kg/m^2^)	21.0	±	1.6		20.8	±	1.6	21.2	±	1.7	21.1	±	1.7	0.894
Resting glucose (mg/dL)	77.1	±	12.5		74.3	±	15.6	80.3	±	12.0	75.7	±	10.0	0.617
Energy and macronutrient intakes during the ultramarathon														
Energy (kcal/kg/h)	2.80	±	2.70		3.31	±	3.64	2.31	±	1.89	3.06	±	1.92	0.072
Protein (g/kg/h)	0.049	±	0.060		0.057	±	0.090	0.041	±	0.040	0.055	±	0.050	0.278
Fat (g/kg/h)	0.057	±	0.090		0.075	±	0.130^a^	0.038	±	0.040^b^	0.068	±	0.080^ab^	**0.043**
Carbohydrate (g/kg/h)	0.48	±	0.43		0.58	±	0.60^a^	0.39	±	0.30^b^	0.48	±	0.24^ab^	**0.040**
Carbohydrate intake (g/h)	26.4	±	9.40		30.6	±	12.8	24.2	±	7.3	24.8	±	7.6	0.384
Mean standardized glucose levels in each segment*														
⊿Average (mg/dL)	32.4	±	14.4		36.0	±	21.2	32.4	±	8.5	28.2	±	13.3	0.645
⊿Highest (mg/dL)	58.4	±	16.2		56.5	±	23.3	63.9	±	12.3	52.4	±	10.0	0.394
⊿Lowest (mg/dL)	12.9	±	13.4		18.6	±	18.7	11.5	±	7.4	8.4	±	13.0	0.382
⊿Highest–lowest (mg/dL)	45.5	±	11.4		37.9	±	8.4^a^	52.4	±	12.7^b^	44.0	±	5.8^ab^	**0.030**
Running speed (%) *^†^	65.8	±	15.8		79.5	±	14.3^a^	59.3	±	9.5^b^	53.7	±	11.5^b^	** < 0.001**

Data are number (categorical variable) or mean ± SD (continuous variables).

Significant difference among the groups was analyzed using Chi-square test (categorical variable) or one-way ANOVA and Tukey's post hoc tests (continuous variables). Values with different letters indicate statistical significance (*p* < 0.05).

*⊿Glucose levels and running speed were standardized as described in Methods.

^†^Kruskal–Wallis test with Bonferroni correction was used due to non-parametric distribution. Significant values are in bold.

As shown in Table S1, habitual energy and macronutrient intakes assessed using BDHQ. One-way ANOVA analyses did not indicate statistical differences in the habitual macronutrient intakes and the ratios among the groups.

### Comparison of energy and carbohydrate intakes

**Figure 2:**
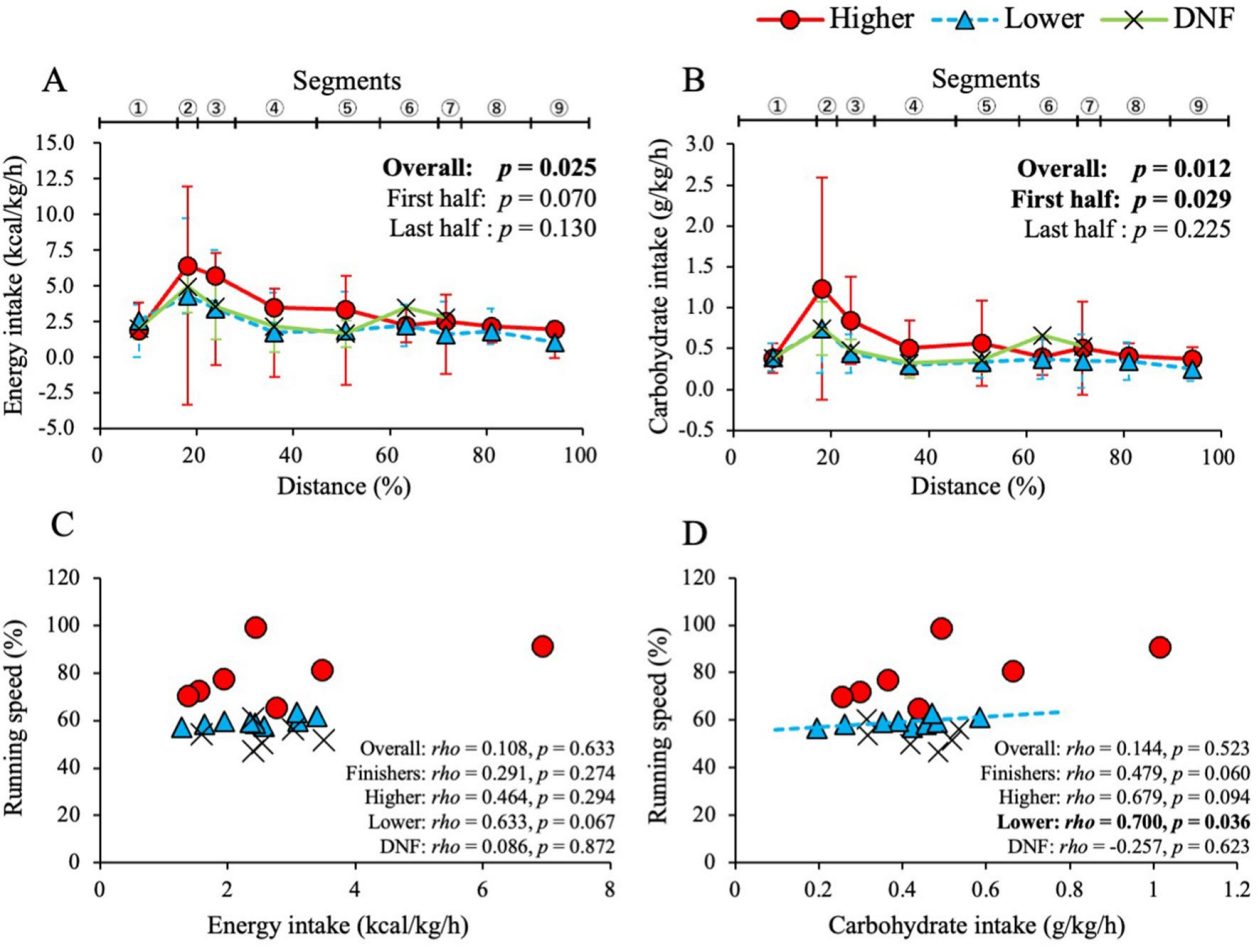
Association of competition results and running speed with energy and carbohydrate intakes during a 100-mile ultramarathon. (**A**) Energy intake among competition results in each segment. (**B**) Carbohydrate intake among competition results in each segment. (**C**) Relationship between standardized running speed and energy intake. (**D**) Relationship between standardized running speed and carbohydrate intake. Red circle (●) indicates data of the higher group, blue triangle (▲) indicates data of the lower group, and cross (×) indicates data of the DNF group. Approximate straight line and the significant correlation were shown as dashed line in the lower group. Boldface indicates statistical significance (*p* < 0.05).

Standardized energy and carbohydrate intakes in each segment were shown in Fig. 2A, B. For finishers, the energy and carbohydrate intakes during the overall segment were significantly higher in higher group than in lower group (*p* < 0.05). When the analysis was divided into the first half (1–5 segments) and the last half (5–9 segments) of the race, energy intakes tended to be higher in the higher group during the first half of the race (*p* = 0.070). The higher group consumed higher carbohydrate intakes than the lower group in the first half (*p* = 0.029) but not in the last half (*p* = 0.225). Although sub-analysis using data from segments 1–3 was performed to compare these dietary intakes among all groups, the results did not indicate significant differences in energy and carbohydrate intakes among the different groups.

### Correlation of running speed with energy and carbohydrate intakes

Standardized energy and carbohydrate intakes of participants during the race were used to analyze the correlation to running speed. As shown in Fig. 2C, there was no significant correlation between running speed and the energy intake in the overall runners. The energy intake of the lower group tended to be positively correlated with running speed (*p* = 0.067). In the lower group, the running speed was significantly and positively correlated with the carbohydrate intake (*p* = 0.036). As shown in Fig. 2D, the carbohydrate intake tended to be positively associated with running speed of higher runners (*p* = 0.094) and finishers, including both higher and lower groups (*p* = 0.060). The DNF group did not show a significant correlation between running speed and dietary intakes during the ultramarathon.

### Comparison of standardized glucose levels

In the DNF participants, the lowest levels of monitoring glucose at last segment were 84.7 ± 14.1 mg/dL, and the ranges were 67–105 mg/dL. The ⊿lowest glucose levels were 9.0 ± 16.9 mg/dL, and the ranges were − 5 to 32 mg/dL.

**Figure 3:**
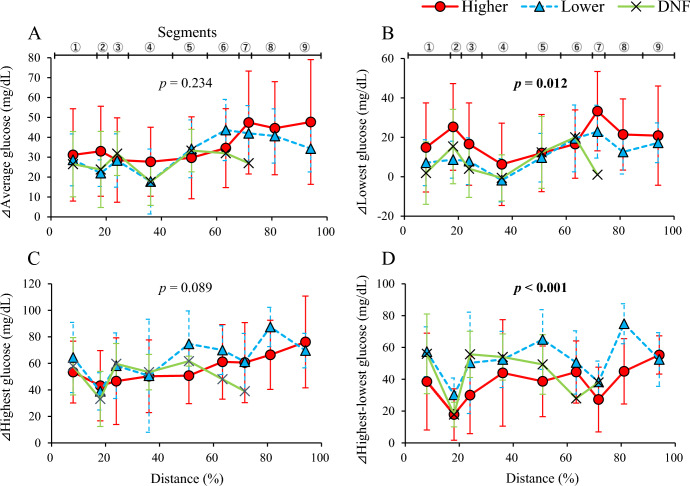
Comparison of standardized glucose levels during a 100-mile ultramarathon. Changes in the glucose levels of ⊿average (**A**), ⊿lowest (**B**), ⊿highest (**C**), and ⊿highest-lowest (**D**) were shown. Red circle (●) indicates data of the higher group, blue triangle (▲) indicates data of the lower group, and cross ( ×) indicates data of the DNF group. P-values indicate results of comparison between higher and lower groups using two-way ANOVA. Boldface indicates statistical significance (*p* < 0.05).

Figure 3 shows changes in ⊿average, ⊿highest, ⊿lowest, and ⊿highest-lowest glucose levels in each segment during the ultramarathon. For finishers, two-way ANOVA analysis revealed significantly higher ⊿lowest glucose levels in the higher group than in the lower group (*p* = 0.012). The lower group showed higher levels of ⊿highest-lowest glucose than the higher group (*p* < 0.001). ⊿Average and ⊿highest glucose levels were not different between the higher and lower groups.

For the sub-analysis using data from segment 1 to 3 among the overall group, the two-way ANOVA analysis found that ⊿lowest glucose levels were significantly different among the groups (*p* = 0.041) and tended to be higher in the higher group than in the lower group (*p* = 0.066) and DNF group (*p* = 0.075) in the post hoc test. Levels of ⊿highest-lowest glucose were significantly higher in the lower group than in the higher group (*p* = 0.023). There were no statistical differences in these standardized glucose levels between the lower group and the DNF group during the first to third segment.

### Correlation between running speed and standardized glucose levels

**Figure 4:**
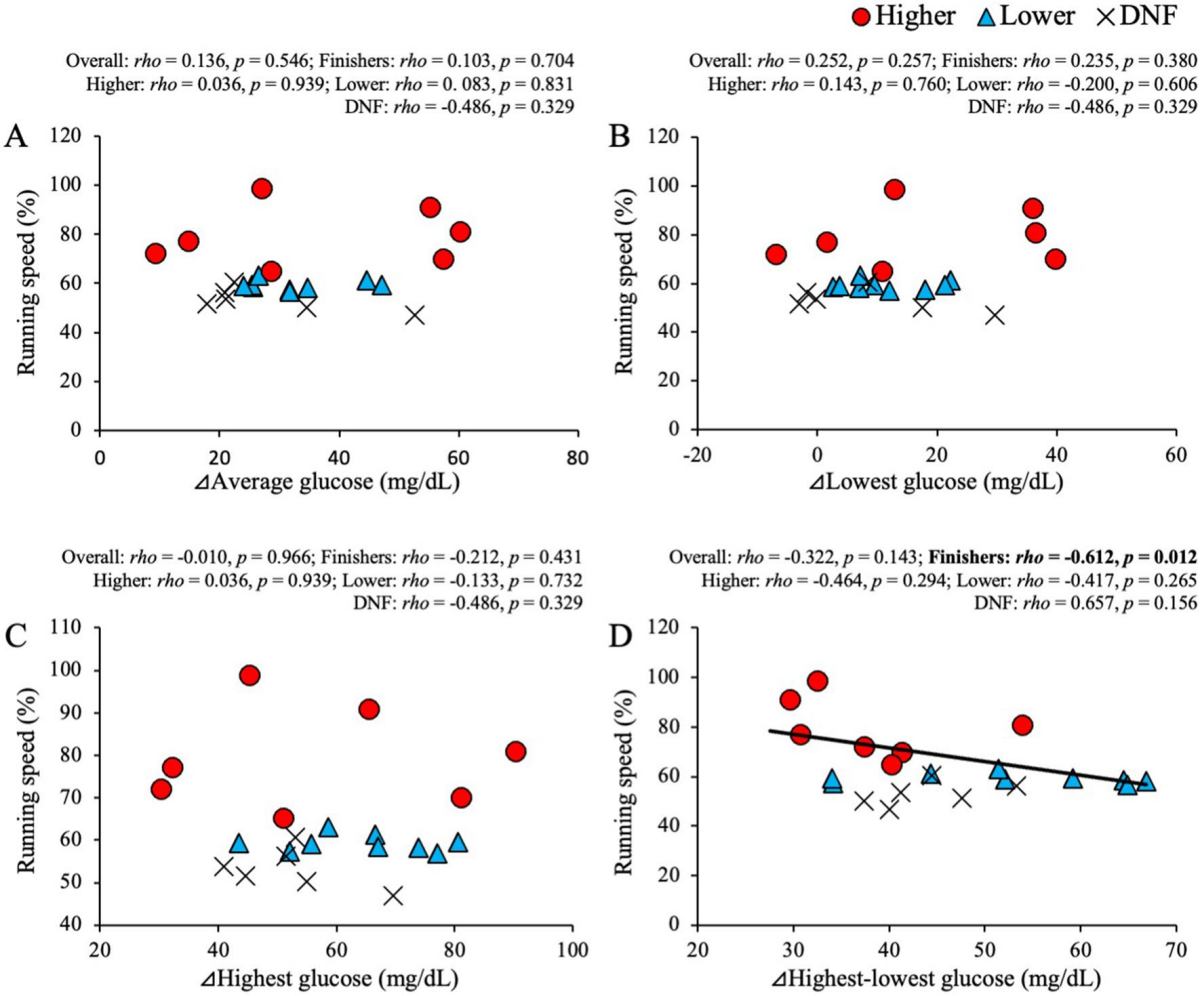
Relationship between standardized running speed and glucose level during a 100-mile ultramarathon. Correlation of the running speed with ⊿average (**A**), ⊿lowest (**B**), ⊿highest (**C**), and ⊿highest-lowest (**D**) were shown. Red circle (●) indicates data of the higher group, blue triangle (▲) indicates data of the lower group, and cross ( ×) indicates data of the DNF group. Approximate straight line and the significant correlation were shown as solid line in the finishers including both higher and lower finishers (*n* = 16). Boldface indicates statistical significance (*p* < 0.05).

A correlation analysis was conducted based on the overall participants, finishers, and among competition results. As shown in Fig. 4, there was no statistical correlation of running speed with ⊿average, ⊿lowest, and ⊿highest glucose levels throughout the race. For the finishers, ⊿highest-lowest glucose levels were significantly and negatively correlated with running speed (*p* = 0.012). The glucose variables were not correlated with running speed in the DNF group.

## Discussion

This observational study reveals the differences in carbohydrate supplementation and glucose homeostasis among competition levels during ultramarathon. Faster runners consume higher amounts of carbohydrates per hour, especially in the first half of the race. In addition, our glucose monitoring during the race found that the fluctuations in glucose levels are associated with running performance in finishers. The results suggest that faster runners pay more attention to carbohydrate supplementation at the beginning of the race, and the maintenance of glucose homeostasis plays a crucial role in ultra-endurance performance.

Previous studies have evaluated the association between physiological factors and running performance in finishers of trail runs^6–8^. However, while running performance was associated with cardiorespiratory fitness levels up to about a 100 km race, the association was not confirmed for longer-distance races^6–8^. The present study compared the differences in nutritional supplementation among different competition levels of a 100-mile ultramarathon and found that nutritional supplementation and glucose homeostasis are associated with the competition results in ultramarathon runners. In the comparison among competition results, the DNF runners were older than the other groups in the present study. It was reported that longer (> 145 km) race performance was correlated with peak velocity during an incremental treadmill test^8^, suggesting that muscle power may contribute to ultra-endurance performance. Skeletal muscle and power decrease with age^27^, and endurance athletes have been reported to have low energy availability, defined as dietary energy intake minus exercise energy expenditure^28,29^. Energy deficiency can cause muscle degradation as a source of energy^30^. Therefore, there is a possibility that the DNF might be associated with decreased running speed due to both aging and energy deficiency.

In this study, the ultramarathon runners did not consume the recommended amount of carbohydrates^17^. This finding is consistent with previous research, which reported that gastrointestinal symptoms are the most common difficulty in following their nutritional strategies^31,32^. Therefore, preventing and addressing gastrointestinal symptoms is crucial for competition results in ultra-endurance athletes. To reduce gastrointestinal symptoms, athletes can undergo "nutritional training," which involves consuming high amounts of carbohydrates during exercise^33^. In order to enhance competition performance, endurance training is essential for improving both aerobic capacity and gastrointestinal function in athletes. Ultramarathon runners utilize various carbohydrate sources, including liquids, gels, fruits, sweets, and solids^12,34^. However, it has not been evaluated which carbohydrate sources are most appropriate for replenishment during ultra-endurance events. Further study is required to examine the optimal supplements on a case-by-case basis. We previously reported that easy to swallow rice cake is a more useful supplement without gastrointestinal symptoms than gels^35^, and thus novel food product is a candidate solution for the symptoms.

Carbohydrates are mainly stored as glycogen in the liver and the skeletal muscle^36^. The liver releases glucose into the blood from glycogen, while the muscle use glycogen as the primary fuel for the contraction^36^. Depletion of these energy sources and subsequent hypoglycemia can significantly slow down running speed, a phenomenon known as "hitting the wall"^37,38^ and may hinder completing the endurance events. Although the study did not determine whether glycogen stores were reduced, the lower levels of ⊿lowest glucose in the lower finishers may suggest an insufficient glucose supply. Throughout the 9 segments of the race, the average levels of ⊿lowest glucose were similar in the middle segments, whereas the levels during the first third of the race were higher in the higher finishers than in the lower finishers and DNF group. It is considered that the first half of carbohydrate intake prevented the decrease in glucose levels in the higher finishers. In addition, ⊿lowest glucose levels were higher during the late third of the race in the higher finishers than in the lower finishers. The mountain elevation was higher in the first and late third of LAKE BIWA 100 (Fig. 1); therefore, it is considered that the high elevation increased the endurance intensity, and thereby glucose levels might be influenced in the lower finishers with lower carbohydrate intake. The subtraction of glucose levels (⊿highest-lowest glucose) was more clearly different between the higher and lower finishers throughout the segments of the race. This is in accord with the results of correlation analysis, which showed that running performance was negatively and significantly associated with ⊿highest-lowest glucose levels in the finishers. The results suggest that fluctuations in glucose levels are a determinant factor for running performance in ultra-endurance events. As mentioned above about related factors of increased and decreased glucose levels, endurance athletes should pay attention to maintaining blood glucose levels during the race.

The present study has several limitations. Firstly, the sample size was relatively small. Secondly, the diet record may be at risk of underestimation, such as under-reporting. Habitual dietary intake was assessed by a BDHQ, whereas a previous study reported that the questionnaire underestimates the absolute value of energy intake^24^ that may be due to unlisted food items and misremembering. The association between habitual diet and endurance performance should be evaluated using more precise measures or appropriate study design. Thirdly, our observational study did not evaluate physiological status such as cardiorespiratory fitness, muscle strength, hydration levels, and glycogen levels. Previous studies reported that cognitive function was associated with ultra-marathon performance^39^; however, we did not evaluate the cognitive changes during the race in the participants, and did not have information of the ergogenic aids such as dietary supplements and pharmacological agents. Because a previous study reported that non-sleepers faster than the sleepers in 100-mile ultramarathon^40^, the data of sleep pattern might be related to the competition results. However, the study did not correct the data of sleep pattern. Sample size of female participants was small, and thus sexual difference could not be analyzed in the present study. These associations among overall determinant factors with running speed during ultramarathon need to be determined in further studies.

## Conclusions

Faster ultramarathon runners consume a high amount of carbohydrates and maintain glucose levels during the race on the trail. Although the key variables were not confirmed for longer-distance ultramarathon, these results indicate the importance of maintaining glucose homeostasis during ultra-endurance competitions. Glucose monitoring during the race and in the training provides how dietary supplementation was beneficial for ultra-endurance performance.

### Supplementary Information

Supplementary Information.

## Data Availability

The datasets used and/or analyzed during the current study are available from the corresponding author on reasonable request.
