# Supplementary material for: A comparative observational study of carbohydrate intake and continuous blood glucose levels in relation to performance in ultramarathon
Source: Sci Rep. 2024 Jan 11;14:1089. doi: 10.1038/s41598-023-51048-6 (PMC10784534; doi:10.1038/s41598-023-51048-6)
Supplement: Supplementary file 1 — Supplementary Information. [file 41598_2023_51048_MOESM1_ESM.docx]

| Table S1. Comparison of habitual dietary energy and macronutrient intakes assessed by a brief self-administered diet-history questionnaire among the groups. | | | | | | | | | | | | | | | |
| --- | --- | --- | --- | --- | --- | --- | --- | --- | --- | --- | --- | --- | --- | --- | --- |
|  |  | Overall (*n* = 22) | | |  | Higher (*n* = 7) | | | Lower (*n* = 9) | | | DNF (*n* = 6) | | | *p* |
| Habitual dietary energy and macronutrient intakes | | | | | | | | |  |  |  |  |  |  |  |
|  | Energy (kcal/day) | 2005 | ± | 631 |  | 2019 | ± | 859 | 2145 | ± | 521 | 1779 | ± | 508 | 0.568 |
|  | Protein (g/day) | 78.5 | ± | 28.5 |  | 77.9 | ± | 38.7 | 85.4 | ± | 25.0 | 69.0 | ± | 20.2 | 0.573 |
|  | Fat (g/day) | 64.4 | ± | 20.9 |  | 56.7 | ± | 22.5 | 70.8 | ± | 23.5 | 63.5 | ± | 13.7 | 0.427 |
|  | Carbohydrate (g/day) | 246.4 | ± | 92.6 |  | 240.1 | ± | 126.0 | 270.5 | ± | 58.6 | 217.7 | ± | 96.8 | 0.567 |
|  |  |  |  |  |  |  |  |  |  |  |  |  |  |  |  |
| Habitual dietary protein, fat, and carbohydrate ratios | | | | | | | |  |  |  |  |  |  |  |  |
|  | Protein (%) | 15.6 | ± | 3.1 |  | 15.1 | ± | 3.4 | 15.9 | ± | 2.5 | 15.9 | ± | 4.0 | 0.869 |
|  | Fat (%) | 29.2 | ± | 6.1 |  | 25.4 | ± | 4.7 | 29.4 | ± | 4.3 | 33.5 | ± | 7.7 | 0.051 |
|  | Carbohydrate (%)^†^ | 48.6 | ± | 7.7 |  | 46.5 | ± | 9.6 | 50.9 | ± | 4.3 | 47.5 | ± | 9.5 | 0.817 |
| Data are mean ± SD. | | | | | | | | | | | | |  |  |  |
| Significant difference among the groups was analyzed using one-way ANOVA and Tukey's post hoc tests (parametric variables) or Kruskal–Wallis test with Bonferroni correction (non-parametric variables). Values with different letters indicate statistical significance (*p* < 0.05). | | | | | | | | | | | | | | | |
| ^†^ indicates non-parametric distribution. | | | | | | | | | | | | | | | |
